# In Vitro and In Vivo Evaluation of Antidiabetic Properties and Mechanisms of *Ficus tikoua* Bur.

**DOI:** 10.3390/nu14204413

**Published:** 2022-10-20

**Authors:** Hanlei Wang, Kun Zhang, Xuelin Chen, Mei Han, Jing Lu, Yumei Zhang

**Affiliations:** 1 Key Laboratory of Tropical Plant Resource and Sustainable Use, Xishuangbanna Tropical Botanical Garden, Chinese Academy of Sciences, No. 88 Xuefu Road, Kunming 650223, China; 2 School of Life Science, University of Chinese Academy of Sciences, Beijing 100049, China

**Keywords:** *Ficus tikoua* Bur., diabetes mellitus, *α*-glucosidase, 3T3-L1 adipocytes, *db/db* mice

## Abstract

In folk medicine, *Ficus tikoua* (*F. tikoua*) has been used to treat diabetes for a long time, but there is a rare modern pharmacological investigation for its antidiabetic effect and mechanisms. Our study aimed to evaluate its hypoglycemic effect using in vitro and in vivo experimental models and then explore the possible mechanisms. In the ethanol extracts and fractions of *F. tikoua*, n-butanol fraction (NBF) exhibited the most potent effect on inhibiting *α*-glucosidase activity (IC_50_ = 0.89 ± 0.04 μg/mL) and promoting glucose uptake in 3T3-L1 adipocytes. Further animal experiments showed that NBF could play an antidiabetic role by ameliorating random blood glucose, fasting blood glucose, oral glucose tolerance, HbA1c level, and islets damage in diabetic mice. Then, the activities of the five subfractions of NBF (NBF1-NBF5) were further evaluated; NBF2 showed stronger *α*-glucosidase inhibition activities (IC_50_ = 0.32 ± 0.05 μg/mL) than NBF. Moreover, NBF2 also possessed the ability to promote glucose uptake, which was mediated via P13K/AKT and AMPK pathways. This study demonstrated that *F. tikoua* possesses antidiabetic efficacy in vitro and in vivo and provided a scientific basis for its folk medicinal use. NBF2 might be potential natural candidate drugs to treat diabetes mellitus. It is the first time the antidiabetic activity and the potential mechanisms of NBF2 were reported.

## 1. Introduction

Nowadays, diabetes mellitus (DM) is one of the most widespread chronic diseases worldwide and seriously affects the quality of human life. Type 2 diabetes (T2DM) is the major type of diabetes, accounting for more than 90% of the total number of patients with diabetes [1]. The pathogenesis of T2DM is insulin resistance or insulin deficiency due to genetic factors, obesity, incorrect components of the diet, aging, and so on [2]. At present, there are many synthetic hypoglycemic drugs with different targets, but due to their high price, limited therapeutic efficacy, or side effects, we are still looking for new drugs to treat diabetes [3]. Herbs have been used to cure diabetes for a long history. Compared with synthetic drugs, plant medicines containing various constituents might reduce blood glucose levels through multiple pathways, thus potentially producing more significant therapeutic effects and fewer side effects.

*α*-Glucosidase is the key enzyme that regulates postprandial blood glucose, which can hydrolyze carbohydrates into glucose in the small intestine. *α*-Glucosidase inhibitors can delay glucose production and absorption to prevent postprandial glucose rise in diabetics [4]. The 3T3-L1 adipocytes are a classical cell line used to research glucose metabolism, insulin signaling, and lipid metabolism in vitro [5,6]. Therefore, the *α*-glucosidase inhibition assay and glucose uptake assay in 3T3-L1 adipocytes were chosen to evaluate the antidiabetic activity of *F. tikoua* in vitro. The *db/db* mice are derived from the autosomal recessive inheritance of the C57BL/KsJ inbreeding strain. They are spontaneously insulin-resistant diabetic mice, which developed symptoms of obesity, hyperglycemia, glycosuria, polydipsia, and polyuria at one month of age [7,8]. The *db/db* mice are a commonly used animal model for researching T2DM in vivo.

*Ficus tikoua* (*F. tikoua*) is a perennial deciduous creeping woody vine, which belongs to the genus Ficus (Moraceae family), and mainly grows in the southern regions of China, including Yunnan, Hunan, Hubei, Guangxi, and other places [9]. It has a long history of being used to treat diseases in folk. In Yunnan district, the water decoction of the dried whole plant is used to treat diabetes, diarrhea, dysentery, and rheumatism [10]. Furthermore, other diseases such as edema, amenorrhea, spermatorrhea, pharyngalgia, and extravasated blood can also be treated with *F. tikoua* [11,12]. Previous phytochemical research reported that *F. tikoua* was not only rich in flavonoids but also contained triterpenoids, steroids, coumarins, organic acids, and other compounds [13,14,15]. Modern pharmacological research of *F. tikoua* was mainly focused on antioxidant [16], antibacterial [17], anti-tumor [18], and antiviral [19] aspects, but there was rare research on its hypoglycemic effect, only one paper reported that the 95% ethanol extract of its rhizome showed *α*-glucosidase inhibitory activity [13]. It is worthwhile to explore the effect and mechanism of *F. tikoua* in treating diabetes.

Therefore, the study is executed aiming to evaluate the antidiabetic potential of *F. tikoua* in vitro and in vivo, providing scientific evidence for its folk medicinal use in treating diabetes. We evaluated the inhibition of α-glucosidase activity and the promotion of glucose uptake in 3T3-L1 adipocytes through in vitro assays and the antidiabetic effect on *db/db* mice for 32 days through in vivo assays. In addition, we investigated the effective fraction of *F. tikoua* with the strongest hypoglycemic activity and explored its possible mechanism of action.

## 2. Materials and Methods

### 2.1. Chemicals and Reagents

*α*-Glucosidase (25.4 Unit/mg), acarbose, and 4-nitrophenyl-*α*-D-glucopyranoside (*p*-NPG) were purchased from Yuanye Biotechnology Co., Ltd. (Shanghai, China). 3T3-L1 mouse preadipocytes were purchased from the American Type Culture Collection (ATCC, Manassas, VA, USA). High glucose DMEM, low glucose DMEM, Pen-Strep solution (P/S), insulin, certified fetal bovine serum (FBS), special newborn calf serum (NBCS), and phosphate-buffered saline (PBS) were purchased from Biological Industries (Shanghai, China). 3-isobutyl-1-methylxanthine (IBMX) and dexamethasone (DEX) were obtained from Sigma-Aldrich (St. Louis, MO, USA). The glucose (glucose oxidase method GOD) kit was purchased from Nanjing Jiancheng Biological Engineering Institute (Nanjing, China). Rosiglitazone (ROSI) was purchased from Meilun Biotech Co., Ltd. (Dalian, Liaoning, China). Insulin, Human Recombinant, was obtained from BioInd (Kibbutz Beit Haemek, Israel). Dimethyl sulfoxide (DMSO) was obtained from Solarbio (Beijing, China). CellTiter 96^®^ AQueous One Solution Cell Proliferation Assay was obtained from Promega Corporation (Madison, WI, USA). The sodium salt of caboxy methyl cellulose (CMC-Na) was purchased from Shanghai Aladdin Biochemical Technology Co., Ltd. (Shanghai, China). Phospho-AMPK*α* (Thr172), PI3 kinase p85, and AS160 antibodies were purchased from Cell Signaling Technology (Danvers, MA, USA). Phospho-PI3K p85 (Tyr458) was purchased from Abmart Shanghai Co., Ltd. (Shanghai, China). Phospho-AKT (Ser473) and AKT antibodies were purchased from Signalway Antibody Co., Ltd (College Park, Maryland, USA). AMPK alpha 1 + AMPK alpha 2 antibody was purchased from Abcam (Cambridge, UK). GLUT4 antibody was purchased from HUABIO Co., Ltd (Hangzhou, China). GAPDH, *β*-actin, secondary anti-mouse, secondary anti-rabbit antibodies, and hypersensitive ECL chemiluminescence detection kit were purchased from Proteintech Group (California, CA, USA). All the chemicals used were analytical grades.

### 2.2. Plant Material

The whole plant of *Ficus tikoua* Bur. was collected in Yuxi, Yunnan, China, in October 2021 and identified by Prof. Yumei Zhang, Xishuangbanna Tropical Botanical Garden, Chinese Academy of Sciences. The voucher specimen (No. 20210723) was deposited in the Innovative Drug Research Group, Xishuangbanna Tropical Botanical Garden, Chinese Academy of Sciences.

#### 2.2.1. Extraction Procedure

The dried *F. tikoua* sample (3.2 kg) was pulverized and extracted by industrial ethanol under reflux three times, then filtered and evaporated under reduced pressure to afford the *F. tikoua* ethanol extract (FEE, 312.0 g). The FEE was dissolved via stirring in hot water and successively partitioned with petroleum ether, ethyl acetate, and n-butanol to afford petroleum ether fraction (PEF, 30.9 g), ethyl acetate fraction (EAF, 46.8 g), n-butanol fraction (NBF, 84.5 g), and aqueous fraction (AF, 149.8 g). Then, NBF was applied to D101 macroporous resin (Tianjin, China) column chromatography and fractioned with ethanol/water (0, 25, 50, 75, and 100%) gradient elution to gain five subfractions (NBF1-NBF5). 

#### 2.2.2. UPLC-ESI-MS Analysis of NBF

The chemical composition of NBF was analyzed using electrospray ionization ultra-performance liquid chromatography-mass spectrometry (UPLC-MS). UPLC system (Agilent, CA, USA) was equipped with A Waters BEH C18 column (100 × 2.1 mm, 1.7 μm), and using the mobile phase with 0.1% formic acid in water (A) and methanol (B) as the following gradient: 0–20 min (16–24% B), 20–22 min (24–26% B), 22–30 min (26–30% B), 30–40 min (30–40% B), and 40–70 min (40–50% B), with the flow rate of 0.3 mL/min. Mass spectrometry (MS) was recorded under the following conditions: ESI source voltage −3.2 kV in the negative ion mode or 4.0 kV in the negative ion mode; sheath gas temperature 350 °C; sheath gas flow 12 L/min; the scan range from 100 to 1500 *m*/*z*. UPLC-MS chromatogram was compared with the Chinese herbal database, and the compounds were identified by the Chinese herbal database’s analytical results. 

### 2.3. In Vitro Study

#### 2.3.1. α-Glucosidase Inhibition Activity

The *α*-glucosidase inhibitory activity was measured according to the procedure described in our previous paper [20]. FEE, PEF, EAF, NBF, and AF were proportioned to different concentrations (20, 40, 80, 160, 320, and 640 μg/mL) with phosphate-buffered saline (PBS). NBF1–NBF5 were proportioned to different concentrations (10, 20, 40, 80, and 160 μg/mL) with PBS. First of all, 80 μL PBS (pH = 6.9) and 50 μL *α*-glucosidase (0.1 Unitu/mL) were added to each well of 96-well plates. Afterward, 10 μL prepared sample solution (20 to 320 μg/mL) was added to each well and mixed; then, the 96-well plates were incubated at 37 °C for 10 min. In the next part, 40 μL *p*-NPG (5 mM) was added to react with the *α*-glucosidase enzyme for 15 min. In the end, 20 μL Na_2_CO_3_ (0.5 M) was added to terminate the reaction. The absorbance value was measured by a microplate reader (Molecular Devices, Palo Alto, Santa Clara, CA, USA) at 405 nm. The *α*-glucosidase inhibition activity was calculated as follows:

Inhibition (%) = [1 − (A_1_ − A_2_)/(A_3_ − A_4_)] × 100 (1)

where A_1_ is the absorbance of the sample group with *α*-glucosidase, A_2_ is the absorbance of the sample blank group without *α*-glucosidase, A_3_ is the absorbance of the control group without sample, and A_4_ is the absorbance of the control blank without *α*-glucosidase and sample. Acarbose was used as the positive control.

The *α*-glucosidase inhibitory activity was indicated by the IC_50_ value calculated via Graphpad Prism 7.0 software (GraphPad Software Inc., San Diego, CA, USA).

#### 2.3.2. Enzyme Kinetic Analysis

The kinetic analysis was performed with reference to previous literature [21,22]. In the test, different concentrations of samples (NBF: 0.0, 0.5, 1.0, and 2.0 µg/mL; NBF2 and NBF3: 0.0, 0.25, 0.5, and 1.0 µg/mL) reacted with five various concentrations of *p*-NPG (0.25, 0.5, 0.75, 1.0, and 1.25 mM), and then the absorbance value was measured at 405 nm. The specific experimental operation was performed as in the *α*-glucosidase inhibition assay. The Lineweaver–Burk plots (the inverse of velocity (1/*v*) versus the inverse of the substrate concentration (1/[S])) were used to determine the inhibition mode of samples. Graphs were drawn using Graphpad Prism 7.0 software.

#### 2.3.3. 3T3-L1 Preadipocytes Culture and Differentiation

Mouse 3T3-L1 preadipocytes were cultured and differentiated using the method described early [23]. 3T3-L1 preadipocytes were cultured in high glucose DMEM supplemented containing 10% NBCS, 1% P/S, and 5% CO_2_ at 37 °C temperature. When the cells achieved 90% confluency, they began to be starved (day 0) for two days. On day 2, the cells were differentiated via culturing into an induction solution (high glucose DMEM supplemented with 10% FBS, 1% P/S, 0.5 mM IBMX, 1 μM ROSI, 1 μM DEX, and 100 nM insulin). On day 5, the cells were cultured in another induction solution (high glucose DMEM supplemented containing 10% FBS, 1% P/S, and 100 nM insulin) for one day (day 6). In the end, the completely differentiated adipocytes were collected for subsequent experiments. 

#### 2.3.4. Glucose Uptake and Cell Viability Assay of 3T3-L1 Adipocytes

The glucose uptake rate was measured as the method described previously [23]. The mature 3T3-L1 adipocytes were inoculated in 96-well plates at the density of 5 × 10^4^ cells per well and cultured in high glucose DMEM medium with 10% FBS for 24 h. Then the medium was replaced with mixed glucose DMEM supplemented (low glucose: high glucose = 3:1) containing 10% FBS, 1% P/S and samples of different concentrations (40 μg/mL, 80 μg/mL, and 160 μg/mL). Insulin (250 ng/mL) was used as the positive control. After 24 h of culture, according to the instructions of the glucose kit, 10 mL medium was taken out and reacted with the reagent for 25 min. The absorbance value was detected by a microplate reader at 505 nm. The glucose uptake rate was calculated as follows:

Glucose uptake rate (%) = [1 − A_2_/A_1_] × 100 (2)

where A_1_ is the absorbance of the mixed glucose DMEM supplemented control, and A_2_ is the absorbance of the blank, insulin, or sample group.

Subsequently, cell viability was measured by CellTiter 96^®^ aqueous cell proliferation test [24], and 15 μL CellTiter 96^®^ AQueous One Solution Cell Proliferation Assay reagent was added to each well and incubated for 8 h at 37 °C. Then, the microplate reader was used to measure absorbance at 490 nm. The relative cell viability was calculated as follows:

Relative cell viability (%) = A_1_/A_2_ × 100 (3)

where A_1_ is the absorbance of the blank control, and A_2_ is the absorbance of the sample group. 

#### 2.3.5. Western Blot Analysis

The differentiated 3T3-L1cadipocytes were inoculated in 24-well plates at 2.0 × 10^5^ cells/well and treated with mixed glucose DMEM supplemented (low glucose: high glucose = 3:1) containing 250 ng/mL insulin, or 40, 80 µg/mL of NBF2 for 4 h. The 3T3-L1cadipocytes were lysed in a mixture of RIPA buffer, phosphatase inhibitors, and protease inhibitors on ice for 30 min to obtain the total protein. The protein concentration was determined using the BCA method. The proteins were separated by electrophoresis using 8% SDS-PAGE gel and then transferred to 0.45 μm PVDF membranes. The PVDF membranes were sealed with 5% skim milk and shaken for 1 h hour. After washing 4 times with TBST buffer, the membranes were incubated with the following primary antibodies overnight at 4 °C: GAPDH (1: 1000), *β*-Actin (1: 1000), AMPK (1: 1000), p-AMPK*α* (1: 1000), PI3K (1: 1000), p-PI3K (1: 1000), AKT (1: 1000), p-AKT, AS160 (1: 1000), GLUT4 (1: 1000), and then washed 4 times with TBST buffer. In the end, the membranes were incubated with the secondary anti-mouse or anti-rabbit antibodies (1: 2500) for 2 h. The protein bands were detected by a hypersensitive ECL chemiluminescence detection kit and quantified by ImageJ software (National Institutes of Health, Bethesda, MD, USA).

### 2.4. In Vivo Study

#### 2.4.1. Experimental Animals and Ethical Statements

Male C57BL/KsJ *db/db* mice (6 weeks old) were acquired from the SiPeiFu (Beijing) Biotechnology Co., Ltd. (Beijing, China). The mice were given one week to acclimatize to the laboratory environment before the formal experiment. The mice were divided into the *db/db* model group and the NBF-treatment group (6 mice/group). The *db/db* model group was given equal volume distilled water (0.5% CMC-Na), and the NBF group was given NBF at a dose of 200 mg/kg body weight by intragastric administration every day. Each group was administered for 32 days. The food intake and body weight were recorded daily. 

This animal experiment was reviewed and approved by the Ethical Committee for the Welfare of Laboratory Animals, Kuming Institute of Zoology, Chinese Academy of Sciences (Ethics review number IACUC-RE-2021-12-004). Try our best to alleviate the suffering of used animals. 

#### 2.4.2. Random Blood Glucose and Fasting Blood Glucose

Random blood glucose and fasting blood glucose were measured once a week on days 0, 7, 14, 21, and 28. The random blood glucose was measured at 8:30 a.m. and then the feed of each cage was removed. After fasting for 6 h (9:00 a.m.–3:00 p.m.), the fasting blood glucose level was measured. All blood samples were collected by the tail tip and surveyed with ACCU-CHEK Performa glucometer (Roche, Germany).

#### 2.4.3. Oral Glucose Tolerance Test (OGTT)

On day 29, the oral glucose tolerance was tested according to the method described in the previous article [25]. Mice that had been fasted for 12 h beforehand were orally administered glucose (2.0 g/kg body weight). Then, blood samples were acquired through the tail tip at 0, 30, 60, 90, and 120 min and measured. After the blood glucose trend curve at 5 time points was drawn, Graphpad Prism 7.0 software (GraphPad Software Inc., San Diego, CA, USA).was used to calculate the area under the curve (AUC).

#### 2.4.4. Insulin Tolerance Test (ITT)

After mice were fasted for 12 h, the insulin tolerance test was implemented [26]. On day 32, all the mice were injected insulin (0.75 Unit/kg body weight) intraperitoneally, and then blood samples were collected at 0, 30, 60, 90, and 120 min through the tail tip. The following manipulations were the same as the above OGTT experiment.

#### 2.4.5. Biochemical Analysis

The whole blood samples were withdrawn from the mice’s eyeballs and separately added into centrifugal tubes with anticoagulant or no anticoagulant before the mice were sacrificed. Successively, serum and plasma samples were obtained by centrifuging at 10,000 rpm for 15 min. The total cholesterol (TC), triglycerides (TG) in serum samples, and glycosylated hemoglobin (HbA1c) in plasma samples were detected using relevant assay kits provided by Kunming Institute of Zoology. CAS (Kunming, China).

#### 2.4.6. Histopathological Analysis

The pathological experiment process was in line with the method described previous paper [27]. After the mice were sacrificed, the whole pancreas and liver in each mouse were removed and fixed in 4% paraformaldehyde and then embedded with paraffin at 65 °C. The hematoxylin-eosin staining (H&E) was used to stain pancreas and liver sections (2–4 μm). The histopathological microscopy and image collection were processed by a Leica DM3000 microscope and its accompanying image acquisition software (Leica, Wetzlar, Germany).

### 2.5. Statistical Analysis

The experimental data were presented as mean ± standard deviation (SD) of three repeated experiments. The significant differences between groups were analyzed by One-way analysis of variance (ANOVA) in vitro and unpaired Student’s *t*-test in vivo with Graphpad Prism 7.0 software (GraphPad Software Inc., San Diego, CA, USA). The differences between groups are considered statistically different at *p* value < 0.05.

## 3. Results

### 3.1. UPLC-ESI-MS Analysis of NBF

**Figure 1:**
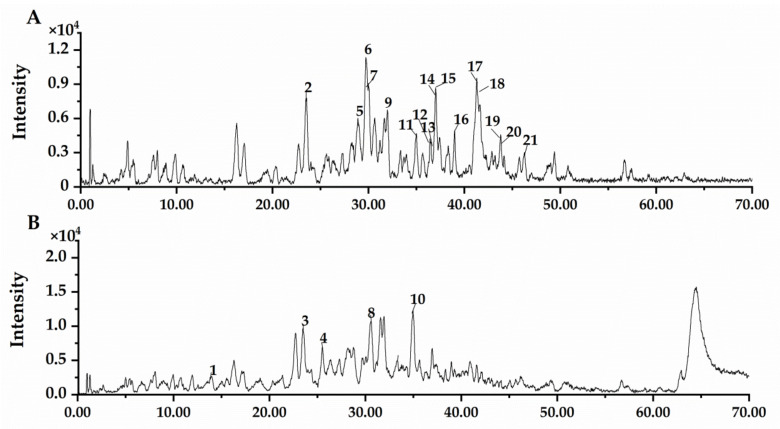
UPLC-ESI-MS total ion current chromatogram of n-butanol fraction of *Ficus tikoua* (NBF). (**A**) Total ion current chromatogram in the negative ion mode. (**B**) Total ion current chromatogram in the positive ion mode.

The components of NBF from *F. tikoua* were tentatively identified by UPLC-ESI-MS. Base peaks of NBF in negative and positive ion modes are shown in Figure 1. Compared with the retention times and ions fragments of compounds in the Chinese herbal database, sixteen compounds of NBF were identified using the negative ion mode and five compounds using the positive ion mode, including seven flavonoids, nine terpenoids, three phenylpropanoids, one steroid saponin, and one quinone. The corresponding phytochemical compounds are exhibited in Table 1.

### 3.2. In Vitro Study

#### 3.2.1. α-Glucosidase Inhibition Activity

*α*-Glucosidase inhibitors can retard carbohydrate absorption by inhibiting the hydrolysis of *α*-glucosidase from reducing postprandial blood glucose levels [28]. For evaluating the antidiabetic property of *F. tikoua*, extract and fractions were examined for their *α*-glucosidase inhibitory activities, and the results are shown in Table 2. The FEE, PEF, EAF, and NBF all exhibited potent inhibitory activities against *α*-glucosidase, except AF (IC_50_ > 32 μg/mL). Among them, NBF had the most potent *α*-glucosidase inhibitory activity (IC_50_ = 0.89 ± 0.04 μg/mL). PEF and EAF showed moderate *α*-glucosidase inhibitory activities (IC_50_ = 2.15 ± 0.09 and 1.60 ± 0.10 μg/mL, respectively). The inhibitory activity of FEE was relatively weak (IC_50_ = 4.46 ± 0.44 μg/mL).

Due to NBF possessing the strongest activity, we further evaluated the inhibitory effects of its five subfractions. We found that NBF2 and NBF3 exhibited the most potent *α*-glucosidase inhibitory activities (IC_50_ = 0.32 ± 0.05 and 0.35 ± 0.03 μg/mL, respectively), which were also superior to NBF. The inhibitory activities of the other subfractions of NBF were relatively weak (NBF1: IC_50_ = 5.23 ± 0.37; NBF5: IC_50_ = 4.28 ± 0.16 μg/mL).

#### 3.2.2. Enzyme Kinetic Study

**Figure 2:**
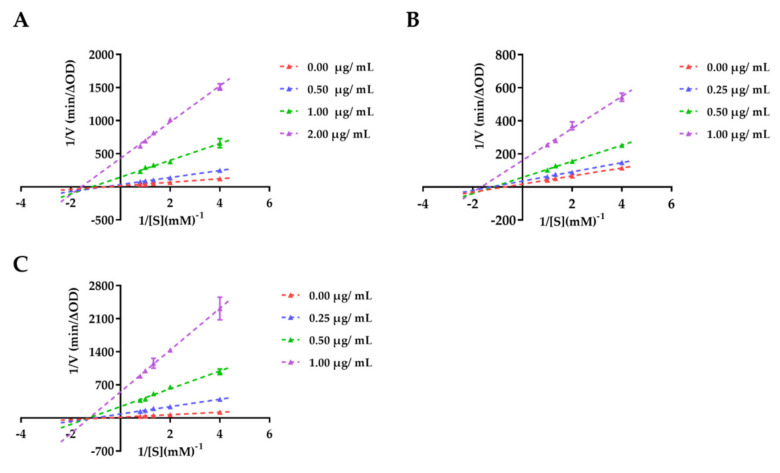
Lineweaver–Burk plots for the inhibition of *α*-glucosidase by NBF, NBF2, and NBF3 using *p*-NPG. (**A**) Lineweaver–Burk plots of NBF on *α*-glucosidase, (**B**) Lineweaver–Burk plots of NBF2 on *α*-glucosidase, and (**C**) Lineweaver–Burk plots of NBF3 on *α*-glucosidase.

NBF, NBF2, and NBF3 were selected to further study for their inhibitory modes because of their potent *α*-glucosidase inhibitory activities. As in the previous literature [22], we analyzed the results by double reciprocal curves. As shown in Figure 2A,B, the lines in the Lineweaver–Burk plots of NBF and NBF2 intersected at the point in the third quadrant, respectively. At the same time, when the concentration of NBF (from 0 to 2.0 μg/mL) and NBF2 (from 0 to 1.0 μg/mL) increased, the Km value (NBF: from 1.80 to 0.64; NBF2: from 1.38 to 0.60) and Vmax (NBF: from 0.067 to 0.002; NBF2: from 0.056 to 0.006) decreased. The results indicated that NBF and NBF2 had a mixed inhibition mode on *α*-glucosidase. As shown in Figure 2C, the lines of NBF3 intersected at the *x* axis, and the Km (1.69) is a fixed value, which implied that NBF3 had a non-competitive inhibition mode on *α*-glucosidase.

#### 3.2.3. Glucose Uptake and Cell Viability of 3T3-L1 Adipocytes

**Figure 3:**
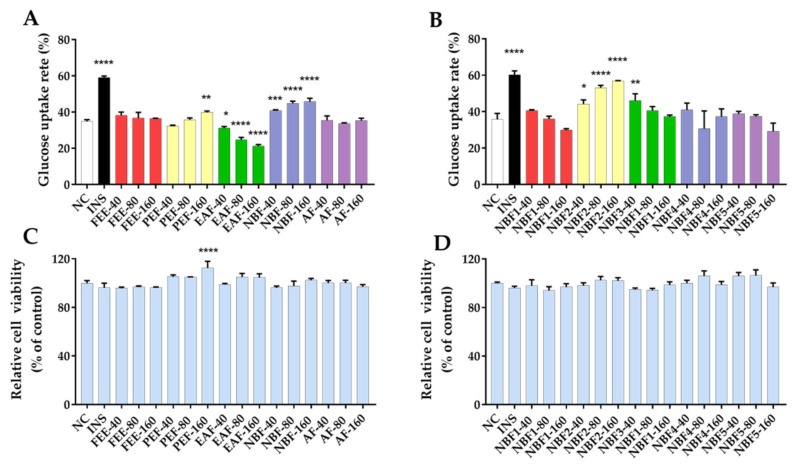
Glucose uptake and relative cell viability in 3T3-L1 adipocytes. (**A**) Glucose uptake rates of *F. tikoua* extract and fractions (FEE, PEF, EAF, NBF, and AF), (**B**) Glucose uptake rates of five subfractions of NBF (NBF1-NBF5), (**C**) Relative cell viability of *F. tikoua* extract and fractions, (**D**) Relative cell viability of five subfractions of NBF. NC: negative control; INS: insulin (positive control), 250 ng/mL; The 3T3-L1 adipocytes were treated with different samples at 40, 80, or 160 μg/mL; Data are presented as mean ± SD (*n* = 3); * *p* < 0.05, ** *p* < 0.01, *** *p* < 0.001, and **** *p* < 0.0001 versus negative control.

In order to assess the antidiabetic ability of *F. tikoua*, we examined the effects of the extract and fractions on glucose uptake in 3T3-L1 adipocytes. As shown in Figure 3A,C, compared with the negative control (NC, 34.90%), NBF significantly promoted glucose uptake in 3T3-L1 adipocytes at 40, 80 and 160 μg/mL (NBF-40: 40.85%, *p* < 0.001; NBF-80: 44.85%, *p* < 0.0001; NBF-160: 45.83%, *p* < 0.0001). In addition, NBF showed a dose-effect relationship in the range of 40–160 μg/mL, and its promoting effect enhanced with the increase in concentration. Compared to the NC group, PEF-160 μg/mL significantly enhanced (*p* < 0.01) glucose uptake, which might be related to the fact that the cell viability of the PEF-160 group was obviously higher (*p* < 0.0001) than that of the NC group. Curiously, the EAF group remarkably inhibited glucose uptake in 3T3-L1 adipocytes at 40, 80, and 160 μg/mL (EAF-40: 31.19%, *p* < 0.05; EAF-80: 24.70%, *p* < 0.0001; EAF-160: 21.26%, *p* < 0.0001) under normal cell viability.

As NBF had the most potent promoting effect on glucose uptake among all the extracts and fractions, a further investigation of its five subfractions was carried out. As shown in Figure 3B, the glucose uptake rates of NBF2 at 40, 80 and 160 μg/mL significantly increased by 28.82% (*p* < 0.05), 48.37% (*p* < 0.0001) and 59.51% (*p* < 0.0001) compared with blank control group, respectively. Furthermore, the effect of NBF possessed a dose–effect relationship in the range of 40–160 μg/mL. Excitingly, the effect of NBF2-160 μg/mL on glucose uptake (56.91%) could approach that of the insulin group (60.13%). The NBF3-40 μg/mL also showed a stimulative effect for glucose uptake (*p* < 0.01). Additionally, the results of cell viability (Figure 3C,D) indicated that all the extract, fractions, and subfractions were not cytotoxic to 3T3-L1 adipocytes compared with the NC group. 

#### 3.2.4. Effect of NBF2 on the PI3K/Akt Pathway, AMPK Pathway and GLUT4 Expression

**Figure 4:**
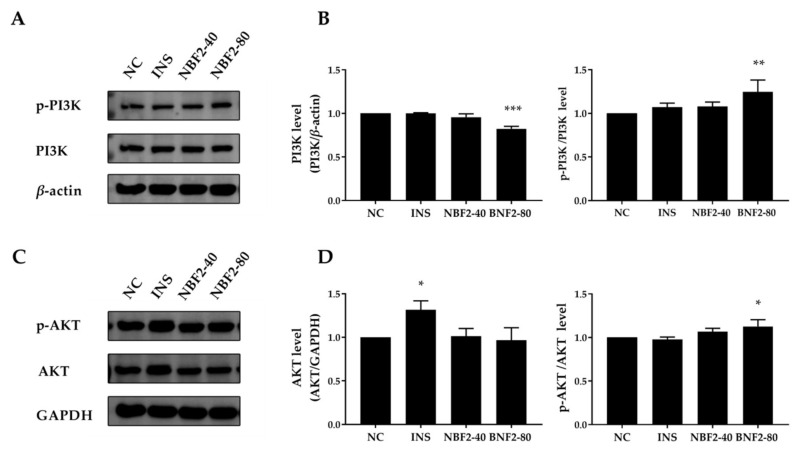
The effects of NBF2 supplementation on p-PI3K (Tyr458), PI3K, p-AKT (Ser473), and AKT protein expression in 3T3-L1 adipocytes. (**A**) p-PI3K (Tyr458) and PI3K protein expression, (**B**) expression levels of PI3K/*β*-actin and p-PI3K/PI3K, (**C**) p-AKT (Ser473) and AKT protein expression, and (**D**) expression levels of AKT/GAPDH and p-AKT/AKT. NC: negative control; INS: 250 ng/mL insulin (positive control); NBF2-40: 40 µg/mL NBF2; NBF2-80: 80 µg/mL NBF2. Data are presented as mean ± SD (*n* = 3). * *p* < 0.05, ** *p* < 0.01, and *** *p* < 0.001 versus negative control.

NBF2 exhibited the strongest promoting effect on glucose uptake in 3T3-L1 adipocytes; thus, we further explored its possible mechanism of action. The expression of *p*-PI3K (Tyr458) and p-AKT (Ser473) proteins can activate the insulin signaling pathway. The insulin signaling pathway plays an important role in glucose uptake. As shown in Figure 4B,D, the PI3K level and p-PI3K/PI3K ratio of INS and NBF2-40 groups were not significantly different from the NC group (*p* > 0.05). Compared with the NC group, the ratio of p-PI3K/PI3K in the NBF2-80 group significantly increased (*p* < 0.01). Meanwhile, the INS group significantly increased the protein level of AKT in comparison with the NC group (*p* < 0.01), whereas the remaining groups were not significantly different from the NC group. The NBF2-80 group significantly upregulated the ratio of p-AKT/AKT compared with the NC group. These results indicated that NBF2-80 might upregulate the expression of p-PI3K and p-Akt, thereby activating the insulin-signaling pathway and improving glucose uptake in 3T3-L1 adipocytes.

**Figure 5:**
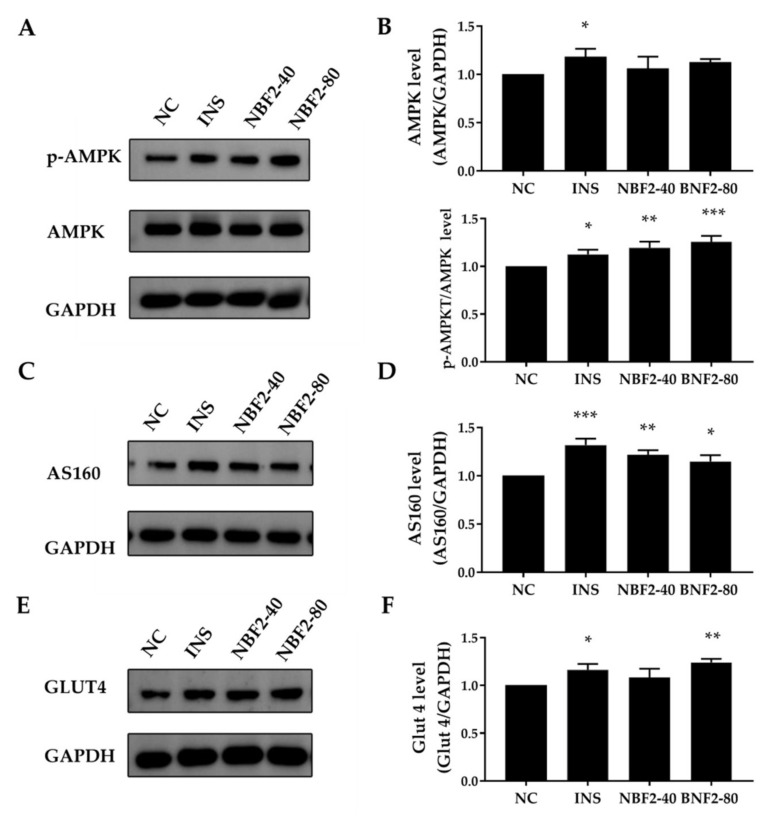
The effects of NBF2 supplementation on p-AMPK*α* (Thr172), AMPK*α*, AS160, and GLUT4 protein expression in 3T3-L1 adipocytes. (**A**) p-AMPK*α* (Thr172) and AMPK*α* protein expression, (**B**) expression levels of AMPK*α*/GAPDH and p-AMPK*α*/AMPK*α*, (**C**) AS160 protein expression, (**D**) expression level of AS160/GAPDH, (**E**) GLUT4 protein expression, (**F**) expression level of GLUT4/GAPDH. NC: negative control; INS: 250 ng/mL insulin (positive control); NBF2-40: 40 µg/mL NBF2; NBF2-80: 80 µg/mL NBF2. Data are presented as mean ± SD (*n* = 3). * *p* < 0.05, ** *p* < 0.01, *** *p* < 0.001 versus negative control.

In addition to PI3K/Akt pathway, the AMPK pathway is also important in glucose uptake. AS160 is a downstream target of AMPK and AKT. As shown in Figure 5B, the protein level of AMPK*α* in the INS group significantly increased compared with the NC group (*p* < 0.05), whereas the remaining groups were not significantly different from the NC group. Moreover, the ratio of p-AMPK*α*/AMPK*α* markedly increased in all intervention groups (INS: *p* < 0.05; NBF2-40: *p* < 0.01; NBF2-80: *p* < 0.001), and the NBF2-80 group exhibited the most significant effect. As shown in Figure 5D, compared to the NC group, the protein level of AS160 significantly increased in INS, NBF2-40 and NBF2-80 groups (*p* < 0.001, *p* < 0.01, *p* < 0.05, respectively). The results showed that NBF2 might promote glucose uptake in 3T3-L1 adipocytes by activating AMPK*α* and upregulating the expression of AS160. GLUT4 is a key protein that transports glucose in adipocytes and muscle cells. The NBF2-80 group significantly upregulated the protein level of GLUT4 in comparison with the NC group (*p* < 0.05; Figure 5F).

### 3.3. In Vivo Study

#### 3.3.1. Effects of NBF on Body Weight and Food Intake

**Figure 6:**
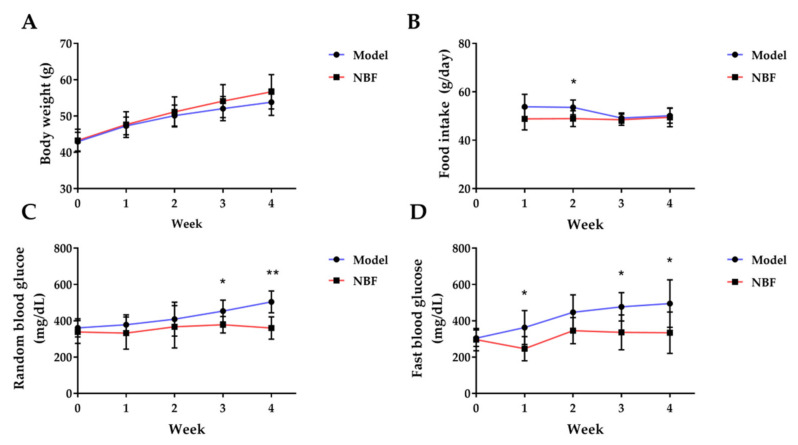
Effects of NBF on body weight, food intake, random blood glucose, and fast blood glucose levels in *db/db* mice during 4 weeks. (**A**) Body weight changes, (**B**) food intake changes, (**C**) random blood glucose changes, and (**D**) fast blood glucose changes. Model: diabetic mice untreated; NBF: diabetic mice treated with NBF at a dose of 200 mg/kg body weight. Data are presented as mean ± SD (*n* = 6); * *p* < 0.05, ** *p* < 0.01 versus model group.

Figure 6A,B show the body weight and food intake of *db/db* mice. The body weight of the model and NBF groups gradually increased during the experiment, but there was no significant difference in weekly body weight between the model group and the NBF group. Compared to the model group, the food intake of the NBF group had no significant difference at 1st, 3rd, and 4th weeks. Only during 2nd week, the NBF group was significantly less than the model group (*p* < 0.05). Overall, it can be assumed that NBF had no effect on body weight and almost no impact on the food intake of *db/db* mice.

#### 3.3.2. Effects of NBF on Random Blood Glucose and Fasting Blood Glucose

The random blood glucose and fasting blood glucose levels of the model group increased continually during the experiment. By comparison, the blood glucose level of the NBF group fluctuated little (Figure 6C,D). The random blood glucose level in the NBF group was significantly lower than that of the model group at the 3rd and 4th weeks and decreased by 16.6% (*p* < 0.05) and 25.5% (*p* < 0.01), respectively (Figure 6C). The fasting blood glucose level of the NBF group was significantly lower than that of the model group at 1st, 3rd, and 4th weeks and decreased by 32.1% (*p* < 0.05) and 29.5% (*p* < 0.05) and 32.4% (*p* < 0.05), respectively (Figure 6D). Thus, the NBF is effective in reducing random and fasting blood glucose levels of *db/db* mice, and the effect on fasting blood glucose is faster than random blood glucose.

#### 3.3.3. Effects of NBF on Oral Glucose Tolerance Test (OGTT) and Insulin Tolerance Test (ITT)

**Figure 7:**
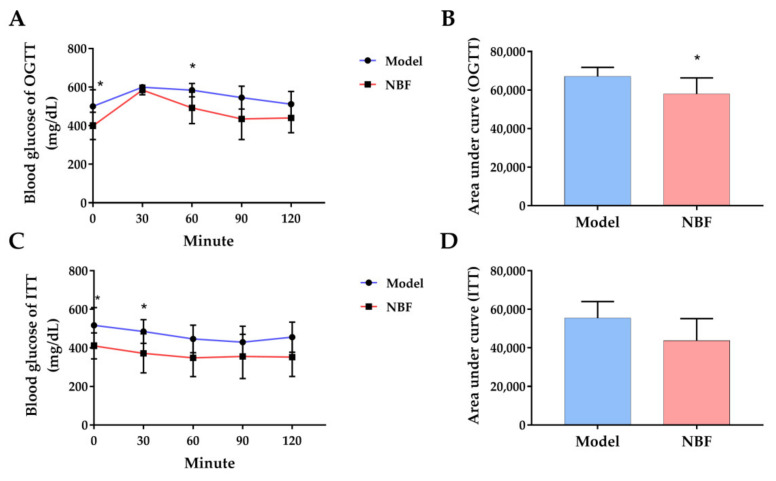
Effects of NBF on OGTT and ITT in *db/db* mice. (**A**) The changes in blood glucose level within 2 h after glucose load, (**B**) the area under the curve of OGTT, (**C**) the changes in blood glucose level within 2 h after insulin injection, and (**D**) the area under the curve of ITT. Model: diabetic mice untreated; NBF: diabetic mice treated with NBF at a dose of 200 mg/kg body weight. Data are presented as mean ± SD (*n* = 6); * *p* < 0.05 versus model group.

The effect of NBF on holistic glucose homeostasis in *db/db* mice was evaluated by OGTT. As shown in Figure 7A,B, the blood glucose levels in the model and NBF groups ascended rapidly to a peak in the first 30 min and then began to descend slowly. However, the NBF group showed faster downward trends than the model group. The blood glucose concentration in the NBF group was lower than that in the model group at 0 and 60 min (*p* < 0.05). Furthermore, compared with the mode group, the area under the curve (AUC) of the NBF group was significantly diminished (*p* < 0.05).

The results of the insulin tolerance test showed that the NBF group had significantly lower glucose levels than the model group at 0 and 30 min (*p* < 0.05; Figure 7C). The AUC of the NBF group had a downward trend compared with the model group, but there was no significant difference between them (Figure 7D).

#### 3.3.4. Effects of NBF on HbA1c, TC and TG Levels

As the related factors of glucose and lipid metabolism, the levels of HbA1c, TC, and TG were detected in this experiment (Table 3). The long-term blood glucose level can be evaluated by Hba1c, which is an important blood glucose indicator. The HbA1c level of the NBF group was significantly lower than that of the model group (*p* < 0.05). The result suggested that NBF could reduce the HbA1c level in *db/db* mice. For the levels of TC and TG, there was no significant difference between the model and NBF groups, indicating that NBF might not ameliorate lipid metabolism disorders.

#### 3.3.5. Effects of NBF on Micromorphology of Pancreas and Liver

**Figure 8:**
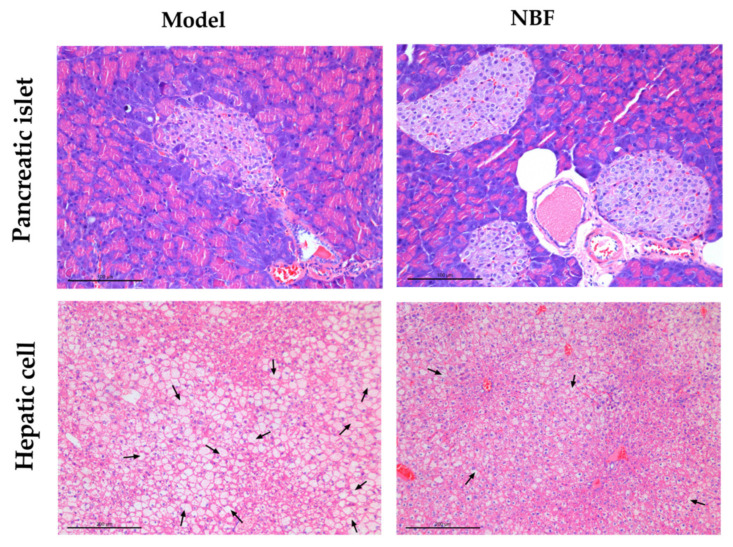
Histopathological section of the pancreas and liver in *db/db* mice. The pancreas (×100 magnification) and liver (×200 magnification) were measured by H&E staining. Model: diabetic mice untreated; NBF: diabetic mice treated with NBF at 200 mg/kg; The arrow points to the extensive vacuolar cells.

We evaluated the pathological changes in the pancreas and liver in each group by H&E staining. As shown in Figure 8, in the model group, the arrangement of islet cells was disordered, in addition, the shape of the islets was irregular, and the margin was incomplete. The treatment of NBF improved islet morphology, and islet cells were arranged more regularly and tightly. For the liver pathological changes, in the model group, hepatic cells were steatotic, and the cytoplasm was filled with lipid droplets. Compared to the model group, the degeneration degree of hepatocytes was slightly improved, and the number of vacuoles was relatively reduced in the NBF group. The results of H&E staining indicated that NBF treatment could ameliorate the damage of the islet and liver in *db/db* mice.

## 4. Discussion

As an herbal medicine, *F. tikoua* has been used to treat diabetes for a long history in the Yunnan region [10]. However, there is no modern pharmacological research to support its hypoglycemic effect and mechanism. In this research, we evaluated its hypoglycemic effect using different experimental models in vitro and in vivo and explored the possible mechanism of action. The results demonstrated that *F. tikoua* possesses a hypoglycemic effect, which may be through inhibiting *α*-glucosidase activity and promoting glucose uptake in adipocytes.

Inhibiting *α*-glucosidase activity is an important way to regulate postprandial blood glucose [29]. Guangmiao Fu et al. found that the 95% ethanol extract of *F. tikoua* exhibited *α*-glucosidase inhibitory activity [13]. Therefore, we firstly evaluated the *α*-glucosidase inhibitory activities of the ethanol extract and four fractions. The results showed the extract and all fractions had inhibitory activities against *α*-glucosidase except AF, among which NBF exhibited the most potent *α*-glucosidase inhibitory activity. The results further proved the validity of the previous research. The UPLC-ESI-MS analysis of NBF showed that it was rich in a variety of flavonoids and terpenoids. A number of previous studies reported that flavonoids have *α*-glucosidase inhibitory activity [29,30,31]; thus, the potent *α*-glucosidase inhibitory activity of NBF might be due to its abundant flavonoids. Then we further evaluated the five subfractions of NBF, among which NBF2 and NBF3 showed the strongest inhibitory activities against *α*-glucosidase. There are four types of inhibitors that inhibit *α*-glucosidase: competitive inhibition, non-competitive inhibition, mixed inhibition, and anti-competitive inhibition [22]. NBF and NBF2 inhibit *α*-glucosidase through a mixed inhibitory mode, and NBF3 inhibits *α*-glucosidase through a non-competitive inhibitory mode. This implies that the active components of NBF and NBF2 may bind both the free enzyme (*α*-glucosidase) and the enzyme substrate complex, whereas NBF3 may bind the enzyme substrate complex.

The glucose uptake model of 3T3-L1 cells is commonly used to study hypoglycemic ability. Recently, existing studies have reported that plant extracts or active components can promote glucose uptake in 3T3-L1 adipocytes to reduce blood glucose [23,32]. The results exhibited that NBF and NBF2 significantly promoted glucose uptake in 3T3-L1 adipocytes with dose-dependent effects, and the effect of NBF2 was more potent than NBF. Our research manifested that NBF and NBF2 could directly ameliorate glucose metabolism in 3T3-L1 adipocytes. Excitingly, NBF2 showed the strongest *α*-glucosidase inhibitory activity as well. It indicated that NBF2 and its components have the potentials to be developed as candidate hypoglycemic drugs.

GLUT4 is transported to the cell membrane to play a critical role in regulating glucose uptake, and PI3K/AKT and AMPK pathways can regulate the translocation of GLUT4 [33]. Therefore, we studied the expression of PI3K/AKT and AMPK pathway-related proteins to explore the possible action pathway of NBF2. Phosphorylated PI3K activates serine/threonine kinase AKT, then phosphorylated AKT eventually lead to phosphorylation of AS160, which triggers the movement of GLUT4 vesicles from cytosol to membrane to regulate glucose uptake in adipocytes [34]. Treatment with NBF2-80 for 4 h increased the phosphorylation of PI3K p85 (Tyr458) and AKT (Ser473), indicating that NBF2-80 had an effect on the insulin pathway. It has been found that flavonoids can activate the PI3K/AKT pathway to promote glucose uptake [35]. Flavonoids were the main type of compounds identified in the UPLC-MS analysis of NBF. Therefore, flavonoids in NBF2 might contribute to the activation of insulin signaling in 3T3-L1 adipocytes. 

AMPK plays a central role in regulating glucose uptake in insulin-independent pathways. Activation of AMPK is achieved by phosphorylation of its Thr172 residue and then phosphorylated AMPK phosphorylates AS160 [36]. We observed that NBF2-80 treatment increased the phosphorylation of AMPK and the expression of AS160. Our study confirmed that NBF2 also might improve glucose uptake in adipocytes through P13K/AKT and AMPK signaling pathways.

The *db/db* mice are commonly diabetic models of spontaneous insulin resistance [8]. To evaluate the hypoglycemic activity of NBF in vivo, a 32-day treatment experiment was conducted in *db/db* mice. In *db/db* mice, the hypoglycemic activity of NBF was evaluated by changes in body weights, food intake, random and fasting blood glucose, OGTT, ITT, and the levels of HbA1c. There was no significant difference in body weight between the model group and the NBF group, which indicated that NBF had no effect on the body weight of *db/db* mice. Compared with the model group, NBF treatment could effectively alleviate the elevation of random and fasting blood glucose in *db/db* mice at the later stage of disease and maintain the relative stability of blood glucose levels. It indicated that NBF possesses dual effects on random blood glucose and fasting blood glucose in diabetic mice, which might be due to its activities of *α*-glucosidase inhibition and glucose uptake promotion. This is different from some plant extracts that only reduce random blood glucose or fasting blood glucose [37,38]. 

OGTT is one of the important indicators to judge the treatment of diabetes. Our study found a significant difference in OGTT between the model group and the NBF group (*p* < 0.05), indicating that NBF could improve glucose tolerance. Improving insulin resistance can increase glucose tolerance [39]. Existing studies showed that flavonoids, terpenes, and phenylpropanoid are the three main natural compounds to improve insulin resistance [40]; thus, the effect of NBF on glucose tolerance may be related to its flavonoids and terpenes. The results of ITT showed that there was no significant difference between the NBF group and the model group, but the NBF group had the tendency to improve insulin tolerance.

Hba1c levels are determined by past, not immediate, blood glucose levels and are not influenced by pre-test fasting, insulin injections, or hypoglycemic drugs. It can reflect the average blood glucose level over the past 8–12 weeks [41]. Compared with the model group, the HbA1c of the NBF group was significantly decreased by about 2% (*p* < 0.05). Research has shown that the risk of diabetes-related death was reduced by 21%, myocardial infarction by 14%, and microvascular complications by 37% when HBA1C decreased by 1% [42]. NBF could reduce mean blood glucose levels, thereby decreasing the likelihood of diabetes complications. 

The islets and liver are crucial target organs for insulin. Islet *β*-cells can produce insulin to maintain glucose homeostasis. Pancreatic islets damage may influence *β*-cells function [43]. When there is insulin resistance or when insulin secretion is insufficient, the anti-lipolysis effect of insulin is antagonized, and sugar utilization is blocked, requiring the mobilization of large amounts of fat, resulting in excessive free fatty acids flowing into the liver. The most common liver injury caused by T2DM is non-alcoholic fatty liver disease, which is a disease state characterized by excessive liver fat deposition [44]. The results indicated that NBF can ameliorate pancreatic islets damage, reduce liver fat accumulation, and thus improve the pathophysiological deterioration of type 2 diabetes mellitus.

These results confirmed the efficacy of *F. tikoua* in treating diabetes. However, there were some limitations in this study. We only evaluated the hypoglycemic effect of *F. tikoua* and explored the mechanisms in vitro. It is not clear what the hypoglycemic mechanisms and exact target in vivo are, and the bioactive compounds of NBF2 deserve further discovery.

## 5. Conclusions

In conclusion, the results suggested that the hypoglycemic potential of *F. tikoua* might be related to the inhibition of *α*-glucosidase activity and the promotion of glucose uptake in adipocytes, and the effect of NBF2 on promoting glucose uptake in adipocytes may be mediated by P13K/AKT and AMPK signaling pathways. Furthermore, the NBF of *F. tikoua* might ameliorate diabetes via reducing fasting and random blood glucose, improving oral glucose tolerance, and decreasing glycosylation of hemoglobin in vivo. Meanwhile, it can alleviate pancreas and liver injury in *db/db* mice. This study explored the hypoglycemic effect and mechanism of NBF2 for the first time and provided a scientific basis for the folk medicinal use of *F. tikoua* to treat diabetes. NBF2 and its active compounds have the potential to be developed as natural candidate plant drugs to cure diabetes. 

## Figures and Tables

**Table 1:** Phytochemical compounds tentatively identified in NBF using UPLC-ESI-MS.

Category	Peak	RT (min)	Identified Compounds	Ionization (ESI^−^/ESI^+^)	*m*/*z*	Molecular Formula	Error [ppm]
Flavonoids	3	23.51	3*S*-(-)-Mucronulatol-7-D- glucopyranoside	[M + H]^+^	465.1739	C_23_H_28_O_10_	−3.5
	4	25.54	Juglanin	[M + H]^+^	419.0956	C_20_H_18_O_10_	−3.96
	5	29.01	6-Hydroxykaempferol-7-*O*- glucoside	[M-H]^−^	463.0887	C_21_H_20_O_12_	1.01
	6	29.73	Equisetrin	[M-H]^−^	609.1465	C_27_H_30_O_16_	0.59
	12	36.45	Apigenin bioside	[M + HCOO]^−^	593.1513	C_26_H_28_O_13_	0.28
	13	36.499	6,8-Bis(C-glucosyl)-apigenin	[M-H]^−^	593.1513	C_27_H_30_O_15_	0.26
	18	41.307	3,5,7,2’,6’-Pentahydroxy flavonol	[M-H]^−^	301.0365	C_15_H_10_O_7_	3.63
Terpenoids	1	14.02	Pteroside D	[M + H]^+^	411.1997	C_21_H_30_O_8_	−4.34
	2	23.49	Pteroside C	[M + HCOO]^−^	441.1776	C_20_H_28_O_8_	2.27
	7	30.07	Zizyvoside I	[M-H]^−^	531.2452	C_25_H_40_O_12_	0.64
	10	34.94	Nigakihemiacetal E	[M + H]^+^	395.2048	C_21_H_30_O_7_	−4.07
	11	35.04	Icariside B9	[M + HCOO]^−^	417.2142	C_19_H_32_O_7_	3.27
	15	37.088	Dendroside F	[M + HCOO]^−^	475.2193	C_21_H_34_O_9_	1.72
	16	39.004	Neohancoside A	[M + HCOO]^−^	493.2296	C_21_H_36_O_10_	0.94
	19	43.755	Gibberellin A8	[M + HCOO]^−^	409.1512	C_19_H_24_O_7_	1.65
	21	46.286	Lucyoside R	[M + HCOO]^−^	711.3959	C_36_H_58_O_11_	0.12
phenylpropanoids	8	30.59	3’-Methoxysecoisolariciresinol	[M + H]^+^	392.1835	C_21_H_28_O_7_	−3.73
	9	31.90	(+)-Medioresinol Di-*O*-*β*-D- glucopyranoside	[M-H]^−^	711.2503	C_33_H_44_O_17_	0.13
	20	43.796	5-*O*-Caffeoyl quinic acid butyl ester	[M-H]^−^	409.1512	C_20_H_26_O_9_	1.51
Steroids	14	37.021	26-*O*-*β*-D-glucopyranosyl(25R)-5*α*-furostane-12-one-3*β*,22 *α*,26-triol-3-*O*-*β*-D-glucopyran	[M + HCOO]^−^	979.4753	C_45_H_74_O_20_	−1.5
Quinones	17	41.274	Purpurin	[M + HCOO]^−^	301.0365	C_14_H_8_O_5_	4.27

**Table 2:** *α*-Glucosidase inhibitory activities of extract and fractions from *Ficus tikoua*.

Samples	*α*-Glucosidase Inhibitory Activity IC_50_ (μg/mL)
FEE	4.46 ± 0.44 ^b^
PEF	2.15 ± 0.09 ^c^
EAF	1.60 ± 0.10 ^d^
NBF	0.89 ± 0.04 ^f^
AF	>32.00
NBF1	5.23 ± 0.37 ^a^
NBF2	0.32 ± 0.05 ^g^
NBF3	0.35 ± 0.03 ^g^
NBF4	1.30 ± 0.08 ^e^
NBF5	4.28 ± 0.16 ^b^
Acarbose	0.01 ± 0.00 ^h^

Data are expressed as the mean value ± SD (*n* = 3); Different letters (^a–^^h^) after the means indicate a significant difference (*p* < 0.05); IC_50_: half inhibition concentration; Acarbose was used as the positive control.

**Table 3:** Effects of NBF on HbA1c, TC, and TG in *db/db* mice.

	Model	NBF
HbA1c (%)	8.84 ± 0.7118	6.52 ± 0.5463 *
TC (mmol/L)	9.82 ± 0.7846	10.09 ± 0.6905
TG (mmol/L)	3.67 ± 0.5936	3.68 ± 0.2363

Data are expressed as the mean value ± SEM (*n* = 5–6); Model: diabetic mice untreated; NBF: diabetic mice treated with NBF at 200 mg/kg. * *p* < 0.05 versus model control.

## Data Availability

Not applicable.
